# Molecular processes underlying the floral transition in the soybean shoot apical meristem

**DOI:** 10.1111/j.1365-313X.2008.03730.x

**Published:** 2008-11-27

**Authors:** Chui E Wong, Mohan B Singh, Prem L Bhalla

**Affiliations:** Plant Molecular Biology and Biotechnology laboratory, Australian Research Centre of Excellence for Integrative Legume Research, Faculty of Land and Food Resources, The University of MelbourneParkville, Vic. 3010, Australia

**Keywords:** floral transition, soybean, shoot apical meristem, hormone, gene expression

## Abstract

The transition to flowering is characterized by a shift of the shoot apical meristem (SAM) from leaf production to the initiation of a floral meristem. The flowering process is of vital importance for agriculture, but the associated events or regulatory pathways in the SAM are not well understood, especially at a system level. To address this issue, we have used a GeneChip® containing 37 744 probe sets to generate a temporal profile of gene expression during the floral initiation process in the SAM of the crop legume, soybean (*Glycine max*). A total of 331 transcripts displayed significant changes in their expression profiles. The *in silico* and RT-PCR analysis on differentially regulated transcripts implies the intriguing involvement of sugar, auxin or abscisic acid (ABA) in events prior to the induction of floral homeotic transcripts. The novel involvement of ABA in the floral transition is further implicated by immunoassay, suggesting an increase in ABA levels in the SAM during this developmental transition. Furthermore, *in situ* localization, together with *in silico* data demonstrating a marked enhancement of abiotic stress-related transcripts, such as trehalose metabolism genes in SAMs, points to an overlap of abiotic stress and floral signalling pathways.

## Introduction

The transition to flowering is a major event in a plant's life that is marked by the switch of the shoot apical meristem (SAM) from leaf production to the initiation of floral organs. Such a developmental transition takes place only when environmental and endogenous factors are most favourable for reproductive success.

One key environmental factor that regulates flowering is the change in day length (photoperiod), as flowering can be controlled by exposure to long days (LDs) or short days (SDs), depending on the plant species (for a review, see Corbesier and Coupland, 2006; Kobayashi and Weigel, 2007). The use of molecular genetic tools in the past decade has dramatically improved our understanding of the molecular aspects associated with the photoperiodic control of flowering time. Studies in the facultative long day (LD) model plant*Arabidopsis thaliana* have revealed that key elements of the LD pathway include light perception and clock components (Corbesier and Coupland, 2006; Imaizumi and Kay, 2006). The interaction of these two components ultimately leads to the circadian rhythm of *CONSTANS* (*CO*) expression, which in turn activates the expression of *FLOWERING LOCUS T* (*FT*): all of this takes place in the leaves. The FT protein has recently been proposed as the much sought-after long-distance signal that travels to the SAM to initiate flowering (Corbesier *et al.*, 2007) by activating the floral meristem identity gene *APETALA1* (*AP1*), together with a bZIP transcription factor (An *et al.*, 2004; Abe *et al.*, 2005). In addition to the LD pathway, other pathways, such as the vernalization or autonomous pathways, are also well characterized in Arabidopsis. Genes such as *LEAFY* (*LFY*), *FT* and *SUPPRESSOR OF CONSTANS1* (*SOC1*) have been identified to be at the junction that integrates inputs from different floral pathways, and conveys the resulting outcome to floral meristem identity genes at the SAM (Parcy, 2005).

Whereas much of our current understanding of the different flowering pathways is derived from the molecular genetic approach focused mostly on Arabidopsis, physiologists were the first to investigate the control of flowering using a variety of plant species (Kobayashi and Weigel, 2007). For example, it was through the grafting experiments performed on several photoperiodic species that the hypothesis of flowering signal(s) transmitted from the leaves to the SAM to initiate flowering was first conceived.

Meanwhile, counterparts of the Arabidopsis flowering-time genes and their targets have been found in a number of other plant species. Although the functional conservation of these orthologs has been demonstrated, there are some intriguing variations on the Arabidopsis theme. For example, the poplar *FLOWERING LOCUS T2* (*FT2*) gene, a relative of the Arabidopsis flowering-time gene *FT*, is not only a part of the flower initiation pathway in poplar, but also plays an additional role in regulating seasonal flower initiation that is integrated with the poplar perennial growth habit (Hsu *et al.*, 2006). Mutant-based functional analyses in pea have shown that the *LFY* ortholog, in addition to its role in floral initiation as reported in Arabidopsis, also plays a part in leaf development, a function not described in Arabidopsis (Hofer *et al.*, 1997). All of these examples demonstrate the ways in which similar basic mechanisms might be adapted to produce the great diversity of plant growth habit.

The flowering process is of vital importance for agriculture and breeding because of its central role in determining crop yield. However, the molecular control of flowering remains very much unknown in the agriculturally and economically important legume species soybean (*Glycine max*). Soybean is one of the world's most important crops, being responsible for 55% of the worldwide oilseed production. The translation of fundamental plant molecular biology knowledge obtained using the model plant Arabidopsis to corresponding processes in legume crop plants remains a challenge, because legume plants display unique vegetative and floral developmental complexities. Both soybean and Arabidopsis are proposed to have diverged from a common ancestor 92 million years ago (Zhu *et al.* 2003). Investigations on gene networks associated with the floral transition in crop legumes such as soybean, and their comparison with the existing knowledge in the model plant Arabidopsis, will not only allow for the identification of evolutionarily conserved processes controlling the floral transition, but will also identify the processes that have undergone independent variation and selection during ∼92 million years of divergent speciation. Soybean in particular offers a quite interesting case because of the availability of individual genotypes that show variability in the photoperiod (and/or temperature) stimulus requirements for the initiation of flowering. Whether the basic flowering pathways revealed from studies in Arabidopsis are conserved in soybean, how the regulation is modified to adjust to the growth habit of a vernalization-unresponsive SD species such as soybean (Summerfield and Roberts, 1983), and what the key to the different maturity groupings in soybean might be remain to be determined.

The current understanding of floral pathways is incomplete, and, as pointed out by Corbesier and Coupland (2006), there is a lack of biochemical components in the pathways. By using other plant species, such as soybean, as model systems to study the flowering process, novel components or networks could be uncovered. Unlike Arabidopsis, soybean floral meristems can revert to leaf production when environmental growth conditions are switched from SDs to LDs (Washburn and Thomas, 2000), and soybean also has a flower development system in which more than one type of organ is initiated at the same time (Tucker, 2003). Information gained from the investigation of the molecular process associated with the floral transition process in soybean will provide a basis to explore these differences in plant development.

We are interested in identifying the molecular events taking place in the soybean terminal shoot apex that leads to the conversion of the SAM into an inflorescence meristem, and the initiation of the floral meristem as a result of an SD photoperiod. Here, we have used a soybean GeneChip® containing 37 744 *G. max* probe sets to study changes in gene expression occurring in the SAM when it converts from a vegetative meristem into an inflorescence meristem. To this end, we isolated RNA from microdissected soybean SAMs at various time points after plants were shifted from non-flowering to flowering-inducing SD growth conditions. We can thus expect to uncover genes that distinguish the SAM before and after floral induction, and genes that are responsible for the initiation of a floral meristem, as well as any potential biochemical processes taking place in the SAM that may account for this switch in the soybean developmental program.

## Results and discussion

### Effect of SD treatment on soybean SAM

**Figure 1:**
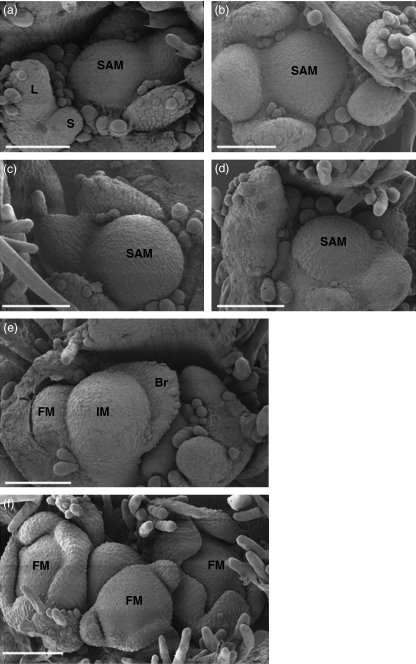
The development of soybean apical meristem. (a, d) Shoot apical meristems of plants grown for 10 (a) or 17 (d) days under long-day conditions with trifoliolate leaves (L) and subtending stipules (S). (b, c, e, f) Micrographs of 10-day-old meristems after experiencing 2 (b; polar view), 4 (c), 6 (e) or 10 (f) short-day cycles, as outlined in the Experimetal procedures. Abbreviations: Br, bract; FM, floral meristem; IM, inflorescent meristem; SAM, shoot apical meristem. Scale bars: 100 μm.

Soybean is a preferential SD plant, i.e. SD growth conditions promote flowering, whereas LD photoperiods prolong vegetative growth (Thomas and Raper, 1983). The cultivar of soybean used in this study is determinate, terminating vegetative activity when the SAM becomes inflorescent. To evoke the floral initiation process in the SAM, soybean plants were grown from seeds under glasshouse conditions (LD photoperiod) for 10 days before shifting to an SD growth chamber (see Experimental procedures). Scanning electron microscopy was then performed on microdissected shoot apices to monitor the morphological changes happening at the SAM in response to different lengths of SD treatment (Figure 1).

On day 10 (0 SD), the SAM is in the vegetative stage of development, with a dome-shaped vegetative apical meristem initiating trifoliolate leaf primordia on its flanks (Figure 1a). After experiencing 2 or 4 SDs the SAMs remain quite similar in their morphology to that at 0 SD, with SAMs at 4 SDs appearing rounder and fuller (Figure 1b,c). Although it was clear that plants had shifted to the reproductive phase of growth after 6 SDs, with the appearance of the first bract of the terminal floral raceme and a floral meristem (Figure 1e), plants of the same age but grown continuously under glasshouse conditions remain vegetative (Figure 1d). Another SEM taken of the SAM after 10 SDs illustrates the continued development of the floral primordium, with a progressively increased number of organ whorls (Figure 1f). Therefore, under our experimental conditions, when plants grown under glasshouse conditions (10-day-old plants) are shifted to an SD growth chamber, the transformation of the SAM from its vegetative stage to the reproductive stage of development is evident morphologically after 6 SDs. We thus decided to use this time point as the end point for our time course experiment, as our primary interest is to map the initial molecular events in the SAM leading to the floral evocation process.

### Identification of differentially expressed transcripts during floral induction

We used the soybean GeneChip® system to monitor the gene expression changes taking place in the SAM during the switch from the vegetative to the reproductive phase of development. RNA was isolated from microdissected soybean SAMs from plants at 10-days old (0 SD), and after 1, 2, 4 or 6 SDs. Whereas only dome-shaped SAMs were dissected for 0, 1, 2 and 4 SDs time points, the newly converted inflorescence meristem and the adjacent floral meristem were collected for the 6-SDs time point, in order to capture transcripts that are essential in establishing the floral meristem.

Raw intensity data from microarray hybridizations were normalized using the Robust Multiarray Averaging method implemented in AffyLimmaGUI (Wettenhall and Smyth, 2004). A methodology called Microarray Significant Profiles (maSigPro) was then used to identify transcripts with significantly different expression profiles in the time-course experiment (Conesa *et al.*, 2006; see Experimental procedures. Unlike statistical methods that focus on pairwise comparison, maSigPro does not evaluate differences between time points, but instead considers the expression profiles across the complete time course, and determines if the corresponding profile for a particular transcript is changing significantly, and hence is effective in capturing the dynamic nature of time-course data (Conesa *et al.*, 2006).

**Figure 2:**
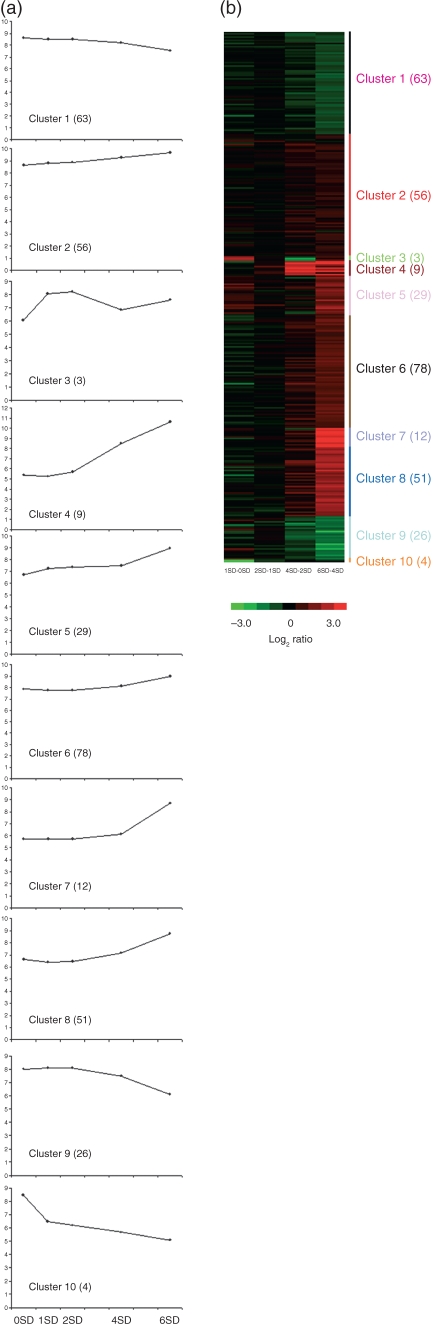
(a) Clustering analysis of 331 transcripts with significant expression profile changes across the time points investigated. The transcripts were classified based on the similarity of their expression profiles using the k-means clustering technique implemented in Cluster 3.0. The *x*-axis indicates the time points at which the shoot apical meristems (SAMs) were dissected following 0, 1, 2, 4 and 6 short days (SDs), whereas the *y*-axis represents the average values of the normalized and log_2_ transformed signal intensity values of the transcripts in the cluster. The total number of transcripts in each cluster is indicated in parentheses. (b) Heat map representing the expression changes for transcripts with differential expression profiles throughout the time points under study.

**Figure 3:**
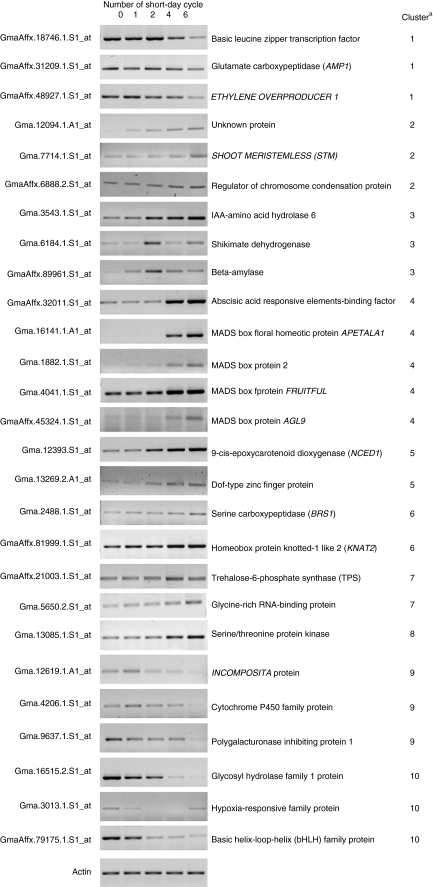
RT-PCR analysis of selected transcripts. RT-PCR analysis was carried out under linear amplification conditions. A transcript annotated as the *actin* gene was used as an internal control. ^a^Clustering was performed using k-means clustering, as described in the Experimental procedures, and the corresponding profile is shown in Figure 2.

Using maSigPro implemented in R, a total of 331 transcripts were detected to have significant changes (adjusted *P* < 0.05) in their expression profiles throughout the time course investigated (Table S1). The 331 transcripts were subsequently clustered according to the pattern of their temporal change in the expression profile into 10 clusters using k-means clustering. The resulting clusters contained transcripts that displayed overall trends of either increased (Figure 2, clusters 2–8) or decreased (Figure 2, clusters 1, 9 and 10) gene expression over the time course of the study. The former set contained a total of 237 transcripts, with members in cluster 4 (Figure 2) displaying the highest -fold changes in gene expression levels. We performed reverse transcription (RT)-PCR analysis on 27 selected genes representative of the 10 clusters to check the validity of the microarray analysis (Figure 3). The soybean actin gene (AFFX-GM_ACTIN_M_AT) has similar intensity values across all data sets, and therefore was used as an internal control. In all cases, the expression profiles obtained by RT-PCR were generally in agreement with those provided by the microarray analysis.

### Transcripts with an increasing trend of expression during floral initiation

#### Induction of putative floral homeotic transcripts

Two thirds of the sequences in cluster 4 are predicted to encode various members of the MADS-box family of transcription factors (Table S1). Among them are *FRUITFUL* (Gma.4041.1.S1_at), *AP1* (Gma.16141.1.A1_at) and *SEPALLATA3* (*SEP3*; GmaAffx.45324.1.S1_at), which were induced drastically after 4 SDs (Figure 2). Similar genes in Arabidopsis are established as floral meristem identity genes, and play important roles in promoting either floral organ formation or inflorescent commitment in floral meristems (Ng and Yanofsky, 2001; Yu *et al.*, 2004). The identified induction of these transcripts in the soybean SAM after 4 SDs reveals that the transition of the soybean SAM to reproductive development has taken place at this particular time point. This also lends further support to the potential of our study to distinguish early molecular events taking place in the SAM that contribute to the progression from the vegetative stage to the inflorescent meristem.

**Figure 4:**
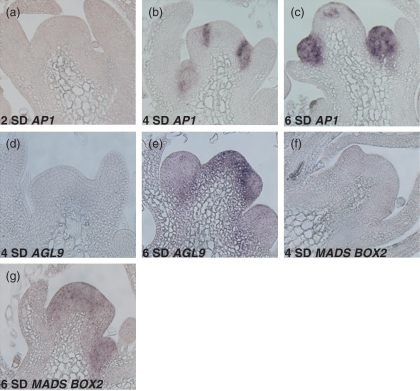
*In situ*localization of putative floral homeotic transcripts in the soybean shoot apices. (a–c) Expression of *APETALA 1* (*AP1*; Gma.16141.1.A_at) was detected after 4 short days (SDs) in the outer region of the shoot apical meristem (SAM), from which the floral primordia eventually emerge. After 6 SDs, a signal was observed in the inflorescent meristem and two adjacent floral meristems. (d, e) Expression of a transcript encoding the MADS-box protein AGL9 (GmaAffx.45324.1.S1_at) was detected after 6 SDs in the main SAM with floral primordia. (f, g) Expression of a putative *MADS BOX 2* gene (Gma.1882.1.S1_at) in the SAM with floral primordia after six SDs. All are longitudinal sections of paraffin-embedded shoot apices probed with the antisense strand of digoxigenin-labelled RNA.

To reveal the spatial expression patterns and to assess the possible conserved function of MADS-box floral genes in soybean, we performed *in situ* hybridization for three selected genes. A robust *AP1*-related signal was apparent in the incipient floral primordia at the floral development stage, with expression of *AP1* throughout the newly emerged floral meristem (Figure 4a–c). Intriguingly, some signal was also observed at the inflorescent meristem after 6 SDs (Figure 4c), which has not been reported in Arabidopsis (Mandel *et al.*, 1992). This probably reflects some divergence of function of *AP1* in soybean. Unfortunately, the accumulation of the other two transcripts after 4 SDs was only detectable by RT-PCR analysis (Figure 3), and not *in situ* (Figure 4d,f). Faint signals associated with these two genes were observed after 6 SDs throughout the inflorescent meristem (Figure 4e,g).

#### Events potentially driving the floral initiation process at the SAM

Clusters 3 and 5 contain transcripts with gene expression induced prior to that of the floral meristem identity genes, i.e. between 0 and 2 SDs. There are three transcripts in cluster 3, and these are annotated as genes encoding indole-3-acetic acid (IAA)-amino acid hydrolase 6 (Gma.3543.1.S1_at), β-amylase (GmaAffx.89961.1.S1_at) or shikimate dehydrogenase (Gma.6184.1.S1_at). Cluster 5 contains 29 transcripts that are predicted to encode a variety of gene products ranging from those that are associated with the phytohormones, auxin, ABA and jasmonic acid, to those that are annotated simply as unknown (Table S1). It is also likely that some important transition events may have occurred between 2 and 4 SDs of treatment, as demonstrated by the marked increase of floral homeotic transcripts in cluster 4.

The differential expression of a putative β-amylase gene (GmaAffx.89961.1.S1_at; cluster 3) is not surprising, as sugar has been suggested to promote the floral transition in many plant species (Bernier *et al.*, 1993; Ohto *et al.*, 2001). The corresponding encoded product is probably associated with the mobilization of reserve starch in the SAM, in response to the changes of relative sink strength that favour the floral transition process. Nonetheless, the availability of sugars could also be an important factor in the control of the expression of a variety of genes, as sugars not only serve as a source of carbon and energy, but also have important signalling functions (Rook *et al.*, 2006). The concomitant upregulation of transcripts associated with sugars and ABA is also in line with the finding that signalling pathways mediated by ABA and sugars interact to regulate plant development (Gibson, 2004; Rook *et al.*, 2006).

In plants, IAA is the form of auxin that generates the majority of auxin effects. Factors that influence the steady-state levels of free IAA in plant cells include biosynthesis by the tryptophan-dependent pathway, and reversible conjugation with amino acids. Our study provides correlative evidence for the early presence of IAA in the SAM before the induction of floral homeotic transcripts. The induction of a putative IAA-amino acid hydrolase 6 (Gma.3543.1.S1_at; cluster 3), as observed in this study (Figure 2), may be associated with the immediate need to hydrolyse the stored IAA-amino acid conjugate to release the active IAA for the floral initiation process. The increased expression of putative tryptophan synthase (Gma.736.1.A1_at), an auxin efflux carrier (Gma.11084.1.S1_at) and a number of auxin-responsive proteins (Gma.17759.1.S1_at, GmaAffx.91936.1.S1_s_at; Gma.2563.1.S1_a_at; Table S1) at a later time point (4 or 6 SDs) further support the possibility that there is an increase in auxin levels in the SAM during the floral initiation process.

**Figure 5:**
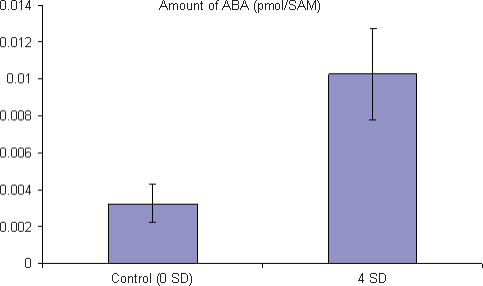
Measurement of ABA levels in the SAM during the floral transition process. Data represent mean values of *n* = 61 plants after 0 short days (SDs) or *n* = 57 plants after for 4-SDs of two biological replicates (levels of ABA: 0 SD_1_ = 0.0025, 4 SD_1_ = 0.012; 0 SD_2_ = 0.004, 4 SD_2_ = 0.0085 pmol per SAM); error bars indicate the ranges of the data.

The 9-cis-epoxycarotenoid dioxygenase (NCED) protein catalyses a rate-limiting step in ABA biosynthesis in plants by cleaving 9-cis-xanthophylls to xanthoxin, a precursor of ABA (Taylor *et al.*, 2005). The upregulation of *NCED1* hints at an increase in ABA levels in the SAM during the floral evocation process. This prompted us to determine whether the increase in ABA biosynthetic transcripts translates to an increase in ABA levels in the SAM. The ABA content in SAMs microdissected from plants treated with 0 or 4 SDs were measured using an immunoassay. There was a significant increase in the ABA level in microdissected SAMs from 4-SD plants in comparison with untreated 10-day-old controls (Figure 5). This correlative evidence implies a floral-promoting role for ABA in soybean. We further compared our data set with the data sets generated from a recent study in Arabidopsis analysing the transcriptional effects of different hormones, including ABA (Nemhauser *et al.*, 2006). This resulted in the identification of 68 transcripts with Arabidopsis orthologues that are responsive to ABA (Table S2). This lends further support to the possibility of an increase in ABA levels in the soybean SAM during the early phase of reproductive development.

**Figure 6:**
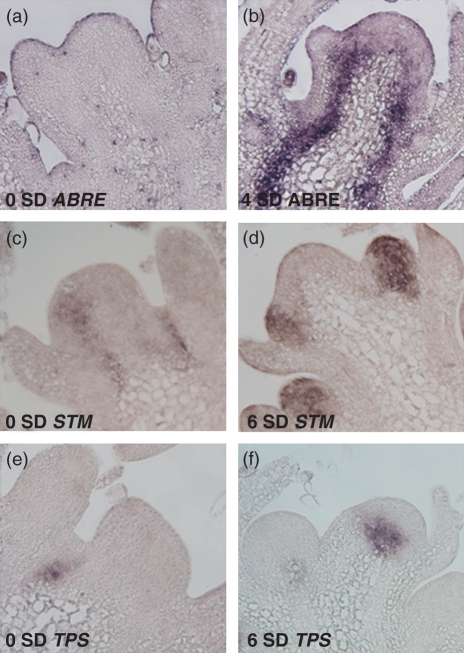
*In situ* localization of putative ABA-related transcript or transcripts associated with the establishment of the floral meristem in the soybean shoot apices. (a, b) Signals related to the expression of an *ABA-responsive element binding factor* (*ABRE*; GmaAffx.32011.1.S1_at) were detected in the region surrounding the vascular system of the shoot apical meristem (SAM) after 4 short days (SDs) (b), but not in that of a vegetative meristem (0 SD; a). (c, d) Expression of the *SHOOT MERISTEMLESS* (*STM*; Gma.15157.1.A1_at) transcript, a meristem regulatory gene, was found in the SAM and in the axillary meristem during the vegetative stage. The transcript is later expressed strongly in the floral meristem, suggesting an important role in vegetative as well as floral meristem development. (e, f) Expression of *TREHALOSE-PHOSPHATE SYNTHASE* (*TPS*; GmaAffx.21003.1.S1_at) was detected in the axillary meristem during the vegetative phase of growth (e). The signal was later detected in the floral meristem (f). All are longitudinal sections of paraffin-embedded shoot apices probed with the antisense strand of digoxigenin-labelled RNA.

Intriguingly, a transcript annotated as *abscisic acid responsive elements-binding factor* (*ABRE*) is grouped together in cluster 4 with other floral meristem identity genes, including *AP1* (Table S1). When the spatial expression of this gene was investigated, signals related to the expression of this gene could be detected surrounding the vascular system at the SAM after 4 SDs, but not in the vegetative SAM (0 SD; Figure 6a,b). This spatial information raises the possibility that the induction of *ABRE* expression may result directly from the arrival of the floral stimulus at the shoot apex, and may subsequently participate in the induction of ABA-responsive genes.

The promoting role of ABA in flowering is ambiguous. A recent study in Arabidopsis has reported an RNA binding protein initially identified for its role in promoting flowering, Flowering time control protein A (FCA), to be an ABA receptor; the interaction of this protein with ABA resulted in a delay of the flowering process (Razem *et al.*, 2006). Arabidopsis mutants with defects in a positive regulator of ABA signalling have been found to display delayed flowering (Brocard-Gifford *et al.*, 2004), which seems to contradict the postulated repressive role of ABA in flowering presented by Razem *et al.* (2006). To complicate things further, there are also Arabidopsis mutants with an impaired negative regulator of ABA signalling, but with an early flowering phenotype (Hugouvieux *et al.*, 2001), and physiological experiments have demonstrated that ABA can promote flowering in a number of SD plants (El-Antably *et al.*, 1967; Podolnyi *et al.*, 1989; Wilmowicz *et al.*, 2008). It is likely that the opposing effects of ABA on flowering revealed from mutant analyses result from pleiotropic effects of the mutations, rather than from direct consequences of altered ABA signalling. FCA is known to act in the autonomous floral pathway; whether this means that ABA may exert a different effect in the photoperiodic regulation of flowering awaits further investigation. Nonetheless, this study has revealed an increase in ABA levels in the SAM during the floral initiation process using an immunoassay, and hence strongly implicates the involvement of ABA in regulating the flowering process.

Our findings raise important questions about the underlying mechanisms by which hormones, ABA in particular, associate with the floral transition process. The induction of hormone-related transcripts in this study occurs prior to known floral genes. There is thus a possibility that these hormones may directly or indirectly regulate the transcription of these genes. For example, the protein farnesyltransferase was found to be involved in ABA signal transduction in Arabidopsis (Cutler *et al.*, 1996), and the Yalovsky *et al.* (2000) study has established that AP1 is a target of farnesyltransferase, and that farnesylation alters the function and perhaps specificity of the transcription factor.

ABA is known as a stress hormone. There was also a striking occurrence of transcripts associated with abiotic stress among the sequences identified to be differentially regulated in the flowering process in soybean. An overlap of the floral signalling pathway in soybean with that of abiotic stress is therefore very probable. Further molecular support comes from a recent study reporting the existence of a cluster of flowering control proteins in the interaction network associated with abiotic stress (Tardif *et al.*, 2007).

There is substantial evidence for crosstalk between the signalling pathways regulating responses to ABA, auxin or sugar. It is likely that each of these is involved in crosstalk and interacts in a complex regulatory network, fine tuning the floral initiation process and thereby allowing SAMs to be flexible in responding to specific developmental and environmental cues.

#### Transcripts associated with the establishment of the floral meristem

Clusters 2, 6 and 7 contain transcripts that are mostly upregulated after 6 SDs (the last time point), with varying magnitudes of increase among the clusters (Figure 2), and these transcripts are likely to function in the establishment of the floral meristem. The presence of a possible meristem regulatory transcript, SHOOT MERISTEMLESS (STM), in cluster 2 probably reflects the increase in meristem number after floral induction. In Arabidopsis, STM is required for the initiation and maintenance of vegetative SAMs (Barton and Poethig, 1993; Endrizzi *et al.*, 1996), in addition to the well-established WUSCHEL-CLAVATA antagonistic pathway (reviewed in Bhalla and Singh, 2006; Singh and Bhalla, 2006). STM is also essential in floral meristem patterning, and in the development of carpels (Scofield *et al.*, 2007). The spatial expression of this transcript throughout the floral meristem seems to suggest that a conserved function is likely (Figure 6c,d). Other transcripts annotated to encode homeobox proteins, such as the KNOTTED1-LIKE family (KNAT2, GmaAffx.81999.1.S1_at; KNAT4, Gma.2376.1.S1_at) in cluster 6, may represent candidates that are likely to be important in the maintenance of the vegetative as well as the floral meristem. In fact, in Arabidopsis, the *KNAT2* (for *KNOTTED*-like from *A. thaliana* 2) homeobox gene is found to be expressed in the vegetative apical meristem, and subsequently plays a role in carpel development (Pautot *et al.*, 2001).

Among the transcripts in clusters 2, 6 and 7, there were three sequences associated with trehalose metabolism (GmaAffx.65216.1.S1.at, GmaAffx.21003.1.S1.at and GmaAffx.5765.1.S1.at; Table S1). Trehalose phosphate synthase (TPS) is known to catalyse the transfer of glucose from uridine diphosphate glucose (UDP) to glucose-6-phosphate, producing trehalose-6-phosphate (T6P) and UDP, whereas trehalose-6-phosphatase (TPP) hydrolyses T6P to release trehalose. When the spatial expression of the two trehalose metabolism-associated transcripts was investigated, we could only successfully detect expression related to TPS (Figure 6e,f). TPS was expressed in the axillary meristem during the vegetative stage of development (0 SD; Figure 6e), and later on, its expression was detected in the centre of the floral meristem (6 SDs; Figure 6f). The presence of *TPS* in the axillary meristem and subsequently in the floral meristem suggests a role in the control of the formation of secondary axes of growth: vegetative and inflorescent branches.

Intriguingly, there is evidence linking ABA with trehalose metabolism (Li *et al.*, 2006), and hence it is tempting to speculate that ABA may act partly through trehalose in triggering the later floral response. Trehalose, a dimer of glucose, is a sugar of emerging significance (Grennan, 2007; Paul, 2007), and has been described as ‘a metabolic regulator that has an impact like that of a hormone’ (Paul, 2007). It is believed that the phoshorylated form of trehalose, T6P, is the active component that regulates its effect. The association of trehalose metabolism with the flowering process was first reported by Dijken and co-workers, who demonstrated in Arabidopsis that TPS is not only essential for normal vegetative growth but also for the transition to flowering (Dijken *et al.*, 2004). A recent study demonstrated that inflorescent branching in maize is determined by a TPP gene, probably through the alteration of a trehalose sugar signal (Satoh-Nagasawa *et al.*, 2006). T6P is associated with the expression of transcription factors that regulate starch synthesis and ABA signalling (Avonce *et al.*, 2004; Ramon *et al.*, 2007), implicating a complex interplay between sugar metabolism and ABA in the floral evocation process in soybean.

### Transcripts with a decreasing trend of expression during floral initiation

The onset of reproductive development is also characterized by the downregulation of genes in incipient floral primordia, and the corresponding transcripts are grouped in clusters 1, 9 and 10. These clusters potentially contain floral repressors that are downregulated to allow floral development to take place. This is exemplified by the downregulation of a transcript annotated to encode the SHORT VEGETATIVE PHASE protein (GmaAffx.9006.2.S1_at). The repression of this MADS-box gene is in agreement with data in Arabidopsis and barley that revealed the corresponding orthologue to be a negative regulator of the flowering process (Hartmann *et al.*, 2000; Trevaskis *et al.*, 2007). The occurrence of this transcript further demonstrates the validity of our data, and strengthens the probability that transcripts in this cluster represent noteworthy candidates for floral repressors for further study.

### Functional categories of transcripts perturbed during floral initiation

As most soybean transcripts could be assigned to a matching Arabidopsis locus, based on its best match with the Arabidopsis protein database, we used GOToolBox (Martin *et al.*, 2004) to identify statistically over-represented gene ontology (GO) terms in our data set to infer functional perturbations in response to floral initiation, or the subsequent stages of floral meristem establishment. When the induced gene data set was analysed with GOToolBox, approximately 60% of the transcripts could be successfully categorized according to GO biological processes (http://www.geneontology.org). Besides the expected GO terms associated with processes such as response to ABA stimulus, regulation of transcription and trehalose metabolism, signal transduction and brassinosteroid-mediated signalling were also significantly enriched in the induced gene data sets (Table 1A). These categories include a two-component phosphorelay mediator (Gma.12547.1.A1_at; AT3G21510), a number of leucine-rich repeat protein kinases, a brassinosteroid (BR) signalling positive regulator, BES1/BZR1 (Gma.1423.1.S1_a_at; AT1G75080) and a serine carboxypeptidase, BRS1 (Gma.2488.1.S1_at; AT4G30610), that plays a role in BR signalling (Li *et al.*, 2001).

**Table 1:** Representative gene ontology (GO) functional categories of the biological process significantly enriched in the (A) induced or (B) repressed gene data set analysed using GOToolBox. The complete GOToolBox output is provided in Table S4

GO identity	Term^a^	*P*-value^b^
A		
GO:0009755	Hormone-mediated signalling	0.0001019
GO:0005991	Trehalose metabolism	0.0002466
GO:0007242	Intracellular signalling cascade	0.000331
GO:0045449	Regulation of transcription	0.0003614
GO:0009742	Brassinosteroid-mediated signalling	0.0003977
GO:0009738	Abscisic acid-mediated signalling	0.000439
B		
GO:0006020	Myoinositol metabolism	0.0001391
GO:0050896	Response to stimulus	0.0004047
GO:0005996	Monosaccharide metabolism	0.0032266
GO:0009725	Response to hormone stimulus	0.0035123
GO:0009910	Negative regulation of flower development	0.0035135

a The GO terms (http://www.geneontology.org) at different depths of the GO hierarchy are given.

b Probability value adjusted for multihypothesis testing using the Benjamini and Hochberg method.

Protein phosphorylation is a key mechanism for intracellular signal transduction in both eukaryotic and prokaryotic cells. The enrichment of protein kinases in the signal transduction functional category prompted us to examine our gene list for the presence of other putative protein kinases that are not annotated using the GO classification system. This resulted in the identification of a total of 15 transcripts annotated to encode different protein kinases (Table 2). In plants, phosphorelay two-component systems play important roles in histidine kinase-mediated signal transduction in response to environmental stimuli and growth regulators (for a review, see Urao *et al.*, 2000). The temporal induction of this kinase in this study suggests it may play additional roles in regulating the flowering process. The induction of other protein kinases, such as the leucine-rich repeat transmembrane protein kinase, may be related to the ABA-mediated signalling pathway, as a recent study has reported a similar gene to be a key membrane-bound regulator of early ABA signalling in Arabidopsis (Osakabe *et al.*, 2005).

**Table 2:** Putative protein kinases induced during the floral initiation process in the soybean shoot apical meristem (SAM)

Probe ID	Cluster	*At* GI^a^	Annotation^a^
Gma.3124.3.S1_a_at	2	AT1G53570	Protein kinase
Gma.7673.1.S1_at	2	AT5G58300	Leucine-rich repeat transmembrane protein kinase
GmaAffx.10790.1.S1_at	2	AT2G36570	Leucine-rich repeat transmembrane protein kinase
GmaAffx.13860.1.S1_at	2	AT5G10290	Leucine-rich repeat transmembrane protein kinase
GmaAffx.5334.1.S1_at	2	AT5G53450	Serine/threonine protein kinase
GmaAffx.86597.1.S1_at	2	AT5G63710	Leucine-rich repeat transmembrane protein kinase
Gma.12547.1.A1_at	4	AT3G21510	Two-component phosphorelay mediator
Gma.10967.1.S1_at	5	AT1G09970	Leucine-rich repeat transmembrane protein kinase
Gma.15630.1.A1_at	6	AT5G56040	Leucine-rich repeat transmembrane protein kinase
Gma.186.1.S1_at	6	AT4G30960	CBL-interacting protein kinase 6 (CIPK6)
GmaAffx.27488.1.S1_at	6	AT4G24480	Serine/threonine protein kinase
Gma.13085.1.S1_at	8	AT4G40010	Serine/threonine protein kinase
Gma.14610.1.A1_at	8	AT3G05140	Protein kinase family protein
Gma.4873.1.S1_at	8	AT4G33950	Protein kinase
Gma.7110.1.S1_at	8	AT1G07650	Leucine-rich repeat transmembrane protein kinase

a Annotation provided by the Harvest website (http://harvest.ucr.edu), and is based on a BLASTX match against the Arabidopsis TAIR database with the matching Arabidopsis gene index, as listed.

Surprisingly, in the repressed gene data set the functional category associated with hormonal response was also significantly enriched (Table 1B). This indicates a complexity of hormone-mediated signalling that involves not only positive but also negative elements. Furthermore, the transcript profiling defining the floral evocation process, or the subsequent stages of floral development described here, involved the gene expression changes of all cell types in the meristem. The up- or downregulation of different genes responsive to the same hormone may reflect the different effects of the hormones on the different cell types in the meristem that are necessary to generate the desired outcome at the SAM.

### Comparison of expression profiles associated with floral transition in Arabidopsis and soybean

Recently, a number of studies have aimed at the characterization of the Arabidopsis floral transcriptome (Schmid *et al.*, 2003; Zhang *et al.*, 2005; Wellmer *et al.*, 2006). However, only the approach of Schmid *et al.* (2003) in Arabidopsis is relevant to this study, as they used excised shoot apexes (i.e. SAMs with leaf primordia and developing leaves) at different time points following floral induction. In contrast, in this study we used microdissected SAMs to obtain a more accurate picture of global gene expression changes happening in the meristem. Changes in the Arabidopsis transcriptome were examined using the Arabidopsis ATH1 array, which contains 22 810 probe sets (Schmid *et al.*, 2003). Using matching Arabidopsis loci assigned to the soybean transcripts (see Experimental procedures), we compared the list of transcripts identified to be differentially regulated in this study with that of Schmid *et al.* (2003) (Table S3).

**Figure 7:**
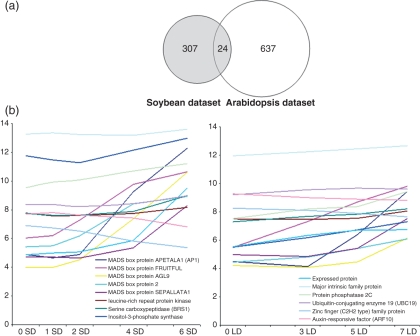
Comparison of the floral data set derived from soybean (this study) with that of Arabidopsis (Schmid *et al.*, 2003). (a) A Venn diagram depicting the overlap between transcripts identified to be differentially expressed in response to the floral evocation process in soybean or Arabidopsis. (b) Expression profiles of orthologous genes in soybean (left panel) or Arabidopsis (right panel).

There was an overlap of only 24 transcripts, with 14 of them displaying similar expression profile changes (Figure 7). The lack of overlap can partly be attributed to the fact that neither the meristem dissection scheme employed nor the time points investigated are exactly equivalent in both studies. Unlike the Arabidopsis experiment, in which shoot apices were used, we used microdissected SAMs. Nevertheless, a closer inspection of the 14 transcripts reveals a high representation of flowering genes (Figure 7), in line with a recent study proposing a conservation of Arabidopsis flowering genes in legumes (Hecht *et al.*, 2005). In addition to the flowering genes, another gene with a similar expression profile in both datasets is *BRS1*, the encoded product of which is associated with BR signalling (Li *et al.*, 2001). BRs have recently been discovered to play unexpected novel roles in the floral induction network (Domagalska *et al.*, 2007).

In summary, our study provides a detailed description of temporal gene expression changes in the soybean SAM undergoing the floral evocation process. We have revealed interesting features and potentially valuable traits associated with the floral initiation process in soybean that may be useful starting points for a more thorough investigation into uncovering novel strategies for manipulating flowering processes, and thus yield, in crop plants.

## Experimental procedures

### Plant materials and RNA extraction

Soybean plants [*G. max*. (L) Merr. Cv. Bragg] were grown from seeds in a glasshouse located at the University of Melbourne, Victoria, Australia, for 10 days (0 SD), before being shifted to a growth chamber under a 10-h light regime (100 μmol m^−2^ s^−1^) maintained at a constant temperature of 24°C. SAMs were microdissected from soybean plants with a 26G syringe needle (Terumo Medical Corporation, http://www.terumomedical.com) under the dissecting microscope at a 40× magnification. Any leaf primordia were excluded in order to create a meristem-enriched tissue collection (Wong *et al.*, 2008). For the sample collection after 6 SDs, the converted inflorescence meristem and the adjacent floral meristem were microdissected, and we consistently observed a similar rate of floral meristem development for all 6-SD replicates. Approximately 80 SAMs were dissected for each time point (0, 1, 2, 4 and 6 SDs). Dissected samples were quickly frozen in liquid nitrogen and stored at −80°C. Total RNA was extracted from dissected SAMs using Qiagen RNeasy Mini Kit with on-column DNAse digestion (Qiagen, http://www.qiagen.com). Two independent tissue collections and RNA extractions were performed for each of the SAM samples. Subsequent cDNA labelling and Affymetrix Soybean GeneChip® hybridization was carried out by AGRF (Australian Genome Research Facility, http://www.agrf.org.au) using 3 μg of total RNA.

### Scanning electron microscopy (SEM)

Soybean shoot apices were dissected under the dissecting microscope at 40× magnification, and were fixed overnight in formalin:ethanol:glacial acetic acid:distilled water (1:5:0.5:3.5). The samples were then dehydrated through an ethanol series, and were stored in 100% ethanol until the process of critical point drying the next day. The samples were dried using a critical point dryer (Model 030; Bal-Tec AG, http://www.bal-tec.com) following the manufacturer's instructions. Dried samples were then carefully placed on a stub before observation using a field-emission scanning electron microscope (FESEM, FEI XL30; Oregon Scientific, http://www.oregonscientific.com).

### Analysis of expression data

Expression levels were estimated from Affymetrix hybridization intensity data using MicroArray Suite 5.0 (Affymetrix 2001, http://www.affymetrix.com). The raw numerical values representing the signal of each feature were filtered so that genes with expression levels referred to as ‘Marginal’ or ‘Absent’ in all 10 arrays according to the Affymetrix statistical algorithms were excluded from further analysis. The resulting data were then normalized using Robust Multiarray Averaging (RMA) implemented in AffylmGUI (Wettenhall and Smyth, 2004), and were then imported into maSigPro, a statistical method employing a two-step regression approach (Conesa *et al.*, 2006). For the first regression model, the *P* value was set at <0.05, whereas in the second regression step a backward method was selected. A combination of quadratic and cubic models was used to evaluate significantly differential expression profiles in the process. Microarray data have been deposited in the GEO database (http://www.ncbi.nlm.nih.gov/geo) under accession number GSE10251.

For the clustering analysis, the log_2_-transformed expression ratios of one time point over the next consecutive time point was computed for the transcripts with significant expression profile changes. Based on these data, transcripts were classified into 10 clusters using k-means clustering analysis implemented in GeneCluster 3.0 (de Hoon *et al.*, 2004), using the Euclidean distance as the similarity metric. The resulting clusters were then visualized using Java TreeView (Saldanha, 2004).

The soybean GeneChip® has been annotated by HarvEST (http://www.harvest-web.org), based on the best BLASTX match of the corresponding soybean sequences against either the TAIR Arabidopsis protein database or the Uniprot protein database. For the comparison of the data set from the analyses of the floral initiation process in Arabidopsis (Schmid *et al.*, 2003), we re-analysed the corresponding data using maSigPro with the same parameters as described above, and the matching Arabidopsis loci for genes of interest were then used for the comparison. Similarly, the matching loci were also used in the search for statistically over-represented GO terms with the program GOToolBox (Martin *et al.*, 2004), using hypergeometric distribution as the statistical test, and Benjamini and Hochberg correction for multi-hypothesis testing (Benjamini and Hochberg, 1995).

### RT-PCR analysis

The one-tube, two enzymes OneStep RT-PCR kit (Qiagen) was used following the manufacturer's instructions in all RT-PCR analyses. RNA isolated from the SAMs at different time points (40 ng) were used as a template in a 10-μl reaction volume. The soybean actin gene (AFFX-GM_ACTIN_M_AT) that showed a similar expression level across the six hybridization experiments was used as an internal control. Routinely, 25–30 cycles were used for the PCR process to ensure that the quantity of amplified product remained in linear proportion to the initial template present in the reaction. The entire PCR reaction was separated on 1% agarose gel containing 0.1 μg μl^−1^ ethidium bromide, and was visualized under UV light.

### In situ hybridization

The soybean shoot apices were dissected from 10-day-old seedlings and fixed with 4% paraformaldehyde (Sigma-Aldrich, http://www.sigmaaldrich.com) in PBS overnight at 4°C after vacuum infiltration. The tissue was then dehydrated and embedded in paraplast (Structure Probe, http://www.2spi.com) following standard methods. The *in situ* hybridization was carried out according to modified protocols from Jackson (1991). Observations and photography were conducted with a microscope BX60 and digital camera DP70 (Olympus, http://www.olympus-global.com). Digoxigenin-labelled antisense RNA probes were transcribed from the T7 or SP6 promoter of pGEM-Easy vector (Promega, http://www.promega.com), using the DIG RNA Labeling Kit (Roche Diagnostics, http://www.roche.com/diagnostics).

### Measurement of ABA level

Soybean SAMs were dissected from plants after 0 or 4 SDs. Two biological replicates of approximately 30 SAMs per replicate per time point were used for the extraction of ABA, following the methods of Xiong *et al.* (2001). The ABA concentration in the extract was then determined using the Phytodetek ABA immunoassay kit (Idetek Inc., http://www.agdia.com/phytodetek).

## Supporting Information

**Table S1.** Transcripts identified to have significant expression profile changes during the floral initiation process in the soybean shoot apical meristem.

**Table S2.** Soybean transcripts with putative Arabidopsis orthologues reported to be responsive to ABA (Nemhauser *et al.*, 2006).

**Table S3.** Arabidopsis transcript identified to be significantly induced or repressed during the floral initiation process; the resulting data set was used in the comparison indicated in Figure 7. The Arabidopsis floral initiation data set was generated by Schmid *et al.* (2003) and has been re-analysed using maSigPro, as outlined in Experimetal procedures, for comparison with the data set in this study.

**Table S4.** Gene ontology (GO) functional categories of the biological process significantly enriched (*P* < 0.01) in the induced (A) or repressed gene (B) data set analysed using GOToolBox (Martin *et al.*, 2004).
